# The non-pathogenic *Escherichia coli* strain W secretes SslE via the virulence-associated type II secretion system beta

**DOI:** 10.1186/1471-2180-13-130

**Published:** 2013-06-12

**Authors:** Mark S DeCanio, Robert Landick, Rembrandt J F Haft

**Affiliations:** 1 Great Lakes Bioenergy Research Center, University of Wisconsin-Madison, Madison, WI, USA; 2 Department of Biochemistry, University of Wisconsin-Madison, Madison, WI, USA; 3 Department of Bacteriology, University of Wisconsin-Madison, Madison, WI, USA

**Keywords:** Type II secretion, Surface display, *Escherichia coli*, Colonization factor

## Abstract

**Background:**

Many pathogenic *E. coli* strains secrete virulence factors using type II secretory systems, homologs of which are widespread in Gram-negative bacteria. Recently, the enteropathogenic *Escherichia coli* strain E2348/69 was shown to secrete and surface-anchor SslE, a biofilm-promoting virulence factor, via a type II secretion system. Genes encoding SslE and its associated secretion system are conserved in some non-pathogenic *E. coli*, including the commonly-used W (Waksman) strain.

**Results:**

We report here that *E. coli* W uses its type II secretion system to export a cognate SslE protein. SslE secretion is temperature- and nutrient-dependent, being robust at 37°C in rich medium but strongly repressed by lower temperatures or nutrient limitation. Fusing either of two glycosyl hydrolases to the C-terminus of SslE prevented it from being secreted or surface-exposed. We screened mutations that inactivated the type II secretion system for stress-related phenotypes and found that inactivation of the secretion system conferred a modest increase in tolerance to high concentrations of urea. Additionally, we note that the genes encoding this secretion system are present at a hypervariable locus and have been independently lost or gained in different lineages of *E. coli*.

**Conclusions:**

The non-pathogenic *E. coli* W strain shares the extracellular virulence factor SslE, and its associated secretory system, with pathogenic *E. coli* strains. The pattern of regulation of SslE secretion we observed suggests that SslE plays a role in colonization of mammalian hosts by non-pathogenic as well as pathogenic *E. coli*. Our work provides a non-pathogenic model system for the study of SslE secretion, and informs future research into the function of SslE during host colonization.

## Background

Gram-negative bacteria use diverse type II secretion systems (T2SS) to deliver a wide variety of proteins into the extracellular milieu [1,2]. Transport is effected by a membrane-spanning complex of 12–15 structural proteins, generically termed Gsp proteins (for general secretory pathway). Secreted substrates first cross the inner membrane by the Sec or Tat pathways; the Gsp proteins then recognize substrates and transport them across the outer membrane. T2SS function requires several proteins that have homologs in type IV pilus biogenesis systems, including an oligomerized secretin, a helical protein filament called the pseudopilus, and a prepilin peptidase essential for pseudopilus assembly [3,4].

Secreted proteins serve many purposes, from electron transport to nutrient acquisition, and some are important pathogenicity factors for plant and animal pathogens in the Enterobacteraceae [5,6]. Type II secretion has been extensively studied in pathogenic strains of *Escherichia coli*, which collectively are known to use two distinct disease-promoting T2SS: the StcE secreting system encoded by the pO157 virulence plasmid [7], and the heat-labile enterotoxin (LT) secreting system common to many pathogenic strains [8]. Recently the latter T2SS was shown for the first time to additionally secrete a non-LT protein, known as SslE, from the enteropathogenic strain E2348/69, thereby promoting biofilm maturation and rabbit colonization by E2348/69 [9,10]. The *sslE* gene sits immediately upstream of the T2SS-encoding secretory genes, and transcription of *sslE* and the *gsp* genes was shown to be co-regulated in *E. coli* strain H10407 [11]. In E2348/69, SslE exists as a lipid-anchored, surface-exposed protein in the outer membrane and is also released into the culture supernatant. Strozen et al. termed the LT- and SslE-secreting system T2SS_β_, to distinguish it from the chitinase-secreting T2SS_α_ that co-occurs in several *E. coli* strains [12]. Based on phylogenetic and structural analyses, Dunstan et al. recently determined that the *E. coli* T2SS_β_ is part of a larger group of T2SS that contain “*Vibrio*-type secretins”, making it a model for numerous type II secretion systems used to deliver toxic substrates by *Vibrio* and *Escherichia* species [10].

The SslE-secreting T2SS_β_, unlike the StcE-secreting pO157 T2SS, is conserved in several non-pathogenic “safe” strains of *E. coli* (“safe” strains may colonize hosts, but have never been known to cause disease), including wild-type B and W isolates [13]. To date, however, no report has described secretion of proteins by T2SS_β_ in any non-pathogenic strain. We were interested to determine whether non-pathogenic *E. coli* could also secrete the “virulence factor” SslE. Secretion of SslE by a safe strain would imply that SslE itself is not capable of promoting a disease state, and would invite comparisons of SslE function between pathogens and non-pathogens. Furthermore, if non-pathogenic *E. coli* could secrete SslE, the T2SS_β_ system could be studied using a non-pathogenic model organism.

We demonstrate here that the non-pathogenic *E. coli* strain W encodes a functional T2SS_β_ that secretes a cognate SslE protein. We found a strong effect of growth conditions on SslE secretion, which is relatively robust in rich medium at 37°C and undetectable when cells are cultured at 30°C or in minimal medium. Previous work suggested that the C-terminus of SslE might be a permissive site for sequence insertions with regards to T2SS_β_ recognition [9], but we found that C-terminal enzyme fusions to SslE blocked protein secretion and surface display.

As noted above, the T2SS_β_ was shown to promote mature biofilm formation in *E. coli* E2348/69. We searched for additional phenotypes in *E. coli* W by phenotypic microarray analysis of a mutant lacking T2SS_β_-encoding genes on Biolog stress plates. The phenotypic microarray indicated a potential fitness effect of the mutation in high concentrations of urea. Using standard culture techniques, we found that deletion of T2SS_β_-encoding genes, or the *sslE* gene, conferred a small survival advantage in medium containing high concentrations of urea.

Our findings make T2SS_β_ the only virulence-associated T2SS with shared functions in pathogenic and non-pathogenic *E. coli*. Considering our regulatory data and the clear homology between the T2SS_β_-encoding operons of W and E2348/69, we propose that SslE is used by non-pathogenic as well as pathogenic strains of *E. coli* during host colonization.

## Results

### E. coli *W secretes SslE using T2SS*_*β*_*under specific temperature and nutrient conditions*

**Figure 1:**
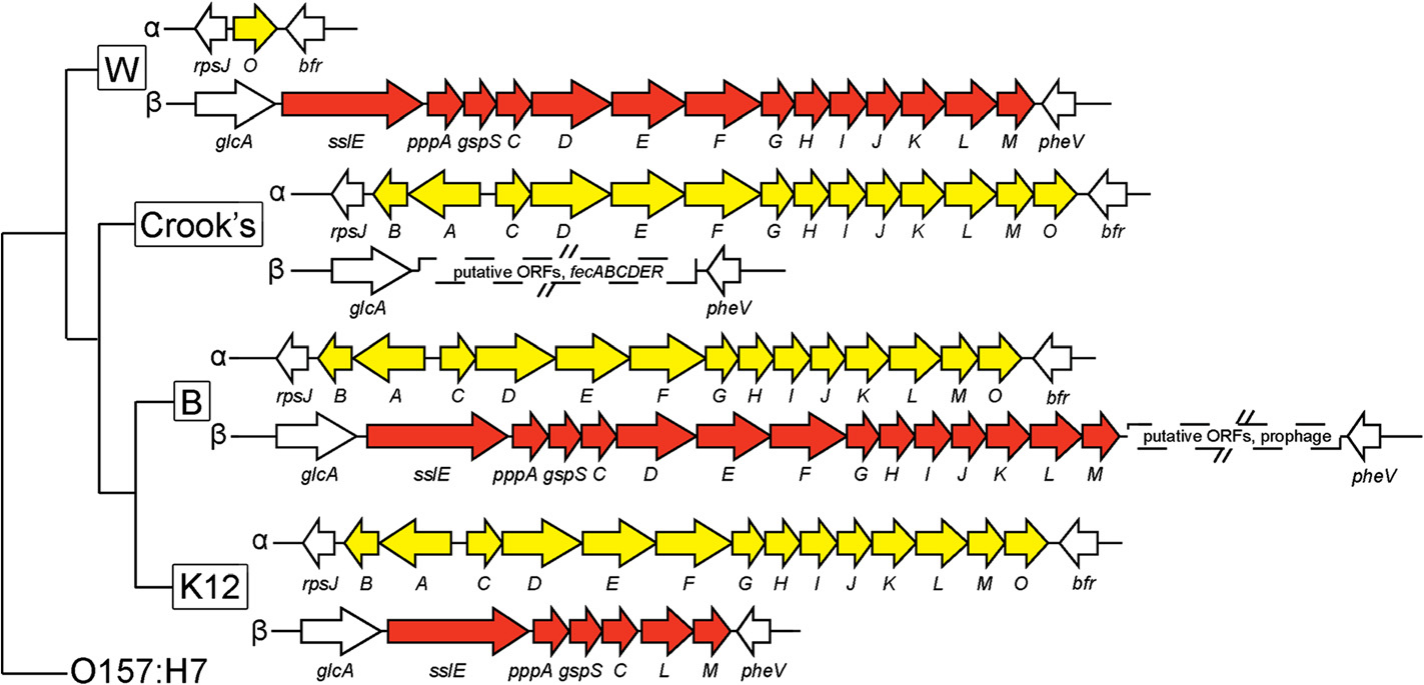
**Distribution of T2SS_α_ and T2SS_β_ in non-pathogenic *E. coli *strains.** Phylogeny is from Archer et al. [13], with O157:H7 as an outgroup lacking both T2SS_α_ and T2SS_β_. Loci encoding the two T2SS types (where present) are diagrammed for each strain. Branch lengths are arbitrary. T2SS_α_*gsp* genes are colored yellow, and T2SS_β_*gsp* genes are shown in red.

**Figure 2:**
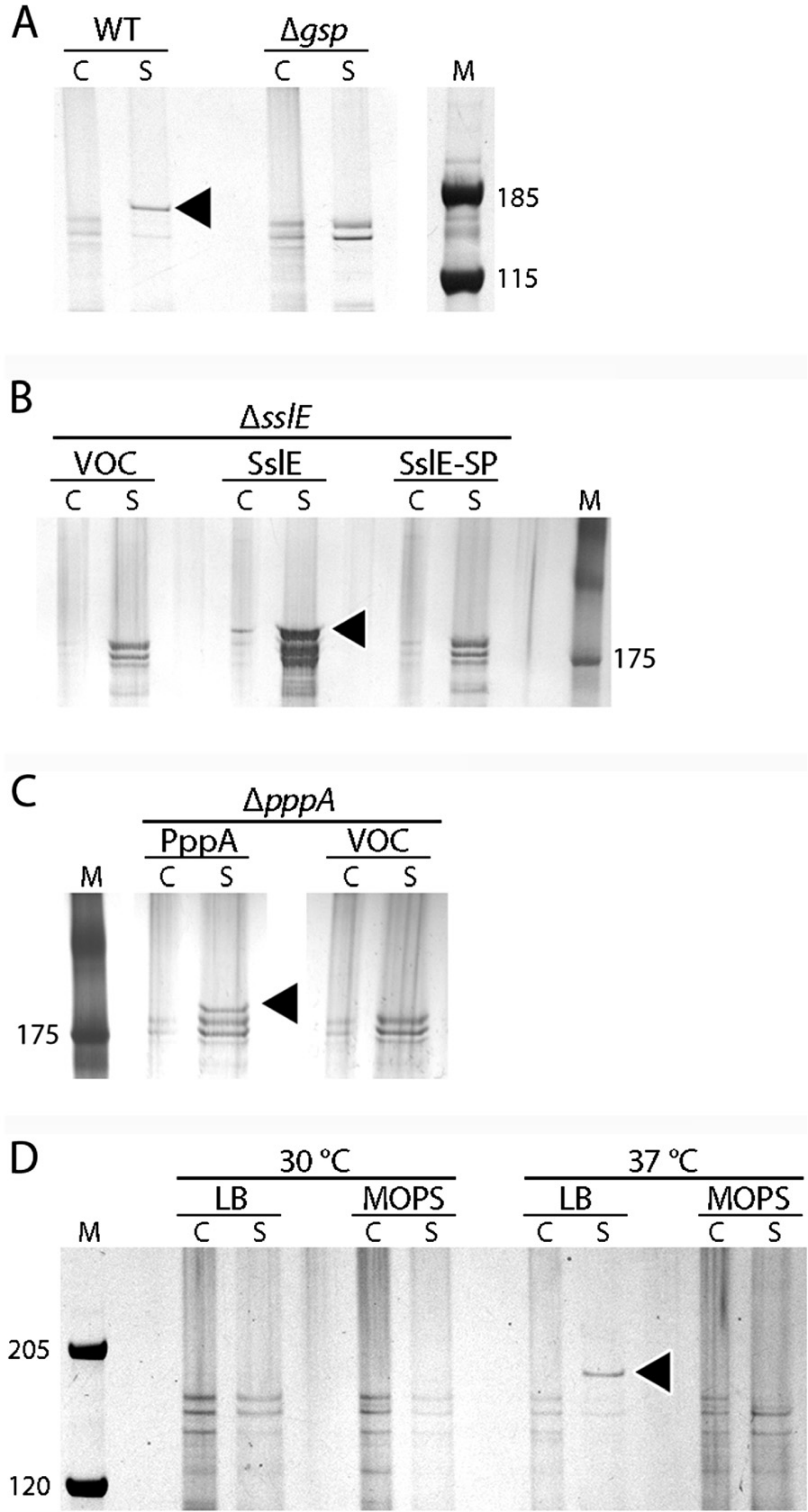
***E. coli *W secretes SslE using T2SS_β_ in a condition-dependent manner.** All lanes are labeled by sample type: C = cell lysate, S = culture supernatant, M = molecular weight standards. **A**. Lysates and concentrated cell-free supernatants of wild-type and Δ*gsp* strains showing SslE secretion by T2SS_β_. **B**. Complementation of the Δ*sslE* mutation: WT = wild-type, VOC = vector-only control, SslE-SP = SslE lacking an N-terminal signal peptide. **C**. Complementation of the Δ*pppA* mutation. **D**. Condition-dependence of SslE secretion labeled by temperature and growth medium. Sizes of molecular weight standards are shown to the side of each gel in kDa. The presence of secreted SslE is marked with black triangles.

Prior to publication of the finished *E. coli* W genome sequence [13], a draft *E. coli* W genomic sequence generated by the U.S. Department of Energy Joint Genome Institute in collaboration with the Great Lakes Bioenergy Research Center (GenBank accession NZ_AEDF00000000) revealed the presence of the entire T2SS_β_ gene cluster, including a copy of the gene encoding SslE (see Figure 1 for a depiction of the locus). To determine whether *E. coli* W secreted endogenous SslE via T2SS_β_, we analyzed the proteomes of the wild-type strain (WT) and a mutant lacking the genes encoding the conserved structural proteins of T2SS_β_ (Δ*gspC-M*). We grew strains in liquid culture, then harvested cells by centrifugation and compared the proteins present in cell lysates and cell-free supernatants (the latter containing any secreted proteins) by SDS-PAGE. We observed a ~180 kDa protein, the expected size for SslE, that was present in the supernatants of WT cultures but not Δ*gsp* cultures (Figure 2A). The ~180 kDa protein band was absent from supernatants and cell extracts of a Δ*sslE* strain, but reappeared when we complemented the *sslE* deletion with plasmid-encoded *sslE* (Figure 2B). To further confirm that SslE was secreted and did not play an intracellular role in activating protein secretion, we attempted to complement the Δ*sslE* strain with a form of SslE lacking the Sec signal peptide (SslE-SP). Unlike wild-type SslE, SslE-SP could not complement the secretory defect in the Δ*sslE* strain. Taken together, our data demonstrate that SslE is secreted from wild-type *E. coli* W by T2SS_β_.

Intracellular SslE did not appear abundant in wild-type *E. coli* W, even under conditions where secretion of SslE was detectable. We observed accumulation of SslE in the cell when SslE was expressed from a multicopy plasmid, however. We postulate that in wild-type cells, the intracellular concentration of SslE is maintained at a relatively low level, and that SslE release from cells over time results in accumulation in the supernatant.

Type II secretion systems require prepilin peptidases to produce the mature, functional forms of their prepilin proteins [1], and the prepilin peptidase PppA is required for secretion of LT by T2SS_β_ in *E. coli* H10407 [12]. To determine whether PppA is similarly required for SslE secretion by *E. coli* W, we compared SslE secretion in WT to a Δ*pppA* strain. SslE secretion was not detectable in the Δ*pppA* background, and the mutation could be complemented by plasmid-encoded PppA (Figure 2C). These results confirm that a fully-functional T2SS_β_ is required to secrete SslE, and indicate that expression of the *gspC-M* genes alone is not sufficient to allow SslE secretion.

We hypothesized that SslE secretion in *E. coli* W might play a role in host colonization, and that secretion might be regulated such that more SslE is secreted under conditions that resemble the mammalian gut. We assessed this conditionality by examining SslE secretion from cultures grown at different temperatures and nutrient conditions: 30°C vs. 37°C, and minimal MOPS-glycerol broth vs. rich LB (Figure 2D). We observed secretion of SslE only in cultures grown in LB at 37°C, indicating that either reduced temperature or nutrient limitations are sufficient to block SslE secretion.

### C-terminal fusions to SslE prevent secretion

In their initial characterization of SslE surface display and secretion, Baldi et al. found that C-terminal fusion of a small tetracysteine-containing motif to SslE did not interfere with localization of SslE [9]. This result suggested that the C-terminus of SslE might not be important for the recognition of SslE by T2SS_β_, and thus might be a permissive site for polypeptide fusions. We were interested in testing C-terminal permissiveness for two reasons: first, because it might provide information about the targeting of SslE for secretion (as there are no defined secretory signals for type II secretion substrates), and second, because SslE fusions might be useful to anchor other proteins to the cell surface. We therefore independently fused two plant cell wall degrading enzymes, Cel45A and Pel10A from *Cellvibrio japonicus*, to the C-terminus of *E. coli* W SslE and assessed the capacity of these fusion proteins to be secreted or displayed on the cell surface. Both fusions resulted in stable, enzymatically active proteins when expressed in *E. coli* W. We did not generate fusions to the potentially lipidated N-terminus of SslE to avoid changes in lipidation that could affect protein localization.

We performed all secretion and display experiments side-by-side in wild-type and T2SS-deficient Δ*pppA* strains, and present the results in Table 1. By following activity of the enzymatic fusions, we found that neither fusion protein was released into the medium under conditions in which we found wild-type SslE to be released. Indeed, extracellular activity of SslE-Cel45A was difficult to detect, though lysed cells released highly active enzyme. Because the substrates for Cel45A (carboxymethyl cellulose) and Pel10A (polygalacturonic acid) are high molecular weight polysaccharides that cannot enter the *E. coli* cell, we were able to assess surface display of fusion proteins by measuring the enzymatic activity of intact cells as compared to cell lysates. These experiments further demonstrated that the fusion proteins were not displayed on the surface of the cell, but accumulated intracellularly.

**Table 1:** Extracellular and surface-displayed activity of SslE-Cel45A and SslE-Pel10A from liquid cultures

** Strain**	**SslE-Cel45A activity**^**a**^		**SslE-Pel10A activity**^**a**^	
	**Supernatant fraction**^**b**^	**Displayed fraction**^**c**^	**Supernatant fraction**^**b**^	**Displayed fraction**^**c**^
WT pRH153	< 0.01	< 0.01	--	--
Δ*pppA* pRH153	< 0.01	< 0.01	--	--
WT pRH154	--	--	0.11(1)	0.08(1)
Δ*pppA* pRH154	--	--	0.10(4)	0.095(5)

^**a**^ Values shown are means of at least two biological replicates, with error in the last digit denoted parenthetically.

^**b**^ Extracellular activity divided by the activity from an equivalent fraction of lysed culture.

^**c**^ Activity measured using intact cells divided by the activity from an equivalent fraction of lysed culture.

### Inactivation of T2SS_β_ modestly increases urea tolerance

**Figure 3:**
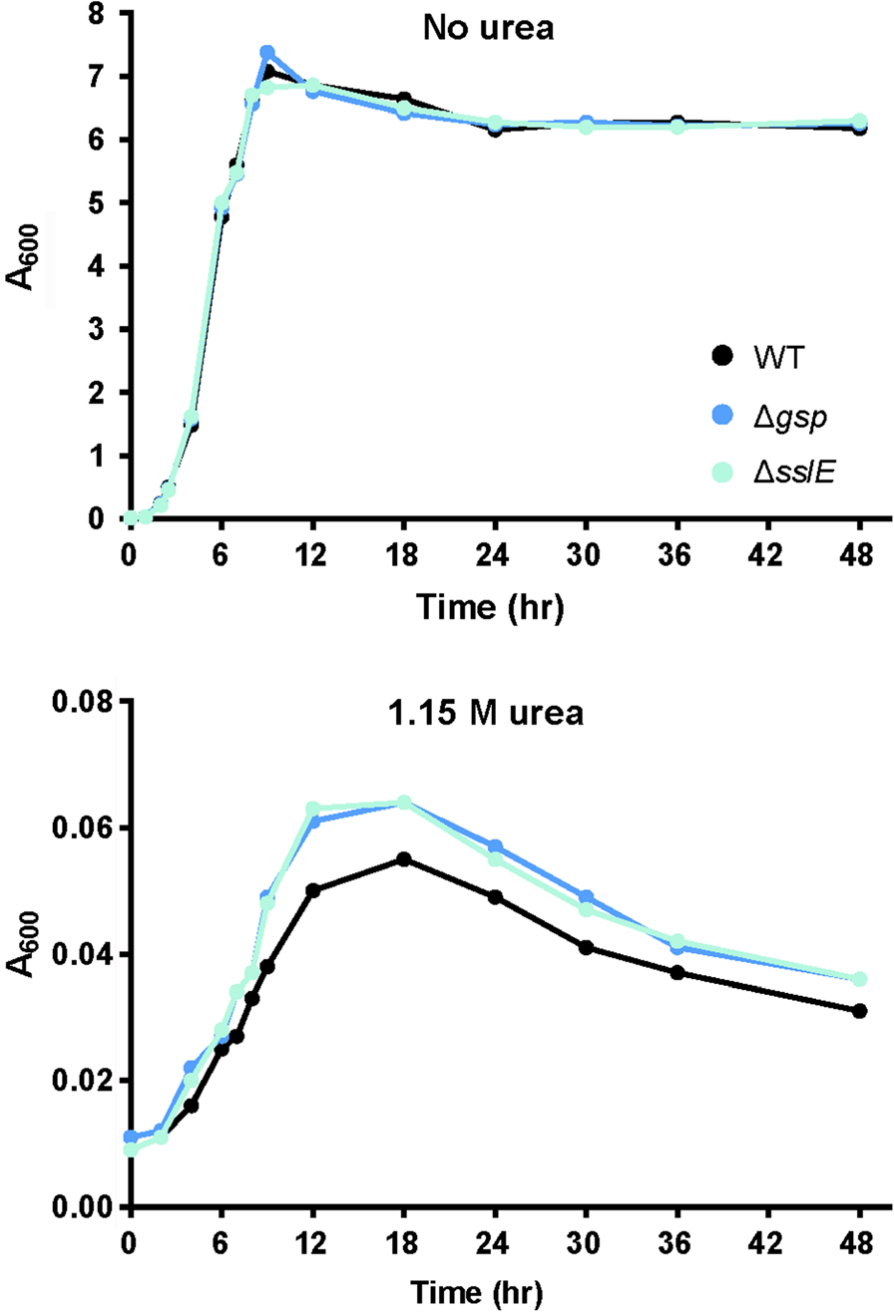
**Growth of wild-type and mutants lacking *gsp *genes or *sslE *with and without urea.** A representative growth curve is shown for each strain grown under the conditions noted.

Baldi et al. demonstrated that inactivation of T2SS_β_ in *E. coli* E2348/69 inhibited biofilm maturation in confocal microscopic analysis of flow cell cultures, though it had no effect on early biofilm development in stationary plate assays [9]. To uncover other phenotypes related to T2SS_β_ disruption, we used *E. coli* W as a non-pathogenic model system in a partial Biolog phenotypic microarray to compare wild-type and Δ*gsp* strains grown with various stressors. The Biolog dye-reduction traces are presented in Additional file 1. Under most conditions the two strains were indistinguishable, but the screen indicated that elevated urea concentrations might differentially affect their growth. We examined this phenomenon in 96-well plate growth experiments under conditions in which our data showed SslE to be secreted (LB at 37°C). Compared to the wild-type control, Δ*gsp* and Δ*pppA* strains maintained higher stationary-phase densities in the presence of 0.90 M and 1.15 M urea (Additional file 2: Figure S1), suggesting that inactivation of the T2SS_β_ system modestly increased urea tolerance even when the structural Gsp proteins were still expressed. We determined the role of SslE in this phenotype and verified modest urea tolerance by following the growth and viability of wild-type, Δ*gsp*, and Δ*sslE* strains for 48 hours with or without 1.15 M urea under the standard culture conditions we used for SslE secretion experiments (in culture tubes on a rolling wheel for vigorous aeration). Culture absorbance readings and viable cell counts indicated that, without urea, the three strains grew equivalently up to 12 hours and slowly lost viability between 12 and 48 hours, with indistinguishable final viable counts at 48 hours (Figure 3 and Table 2). In the presence of 1.15 M urea all strains grew poorly, but Δ*gsp* and Δ*sslE* strains maintained higher turbidity and viable cell counts than wild-type, with both mutants having > 60% more surviving cells than wild-type at 48 hours. We conclude that the inability to secrete SslE confers a small survival advantage in the presence of high concentrations of urea.

**Table 2:** Viable cell counts for cultures grown with and without urea

** Strain**	**Urea**^**a**^	**6 hr**^**b**^	**12 hr**^**b**^	**24 hr**^**b**^	**48 hr**^**b**^
Wild-type	–	2.8 ± 0.1 × 10^9^	6.9 ± 0.3 × 10^9^	2.0 ± 0.3 × 10^9^	1.2 ± 0.1 × 10^9^
Δ*gsp*	–	2.6 ± 0.3 × 10^9^	6.2 ± 0.2 × 10^9^	2.4 ± 0.2 × 10^9^	1.2 ± 0.1 × 10^9^
Δ*sslE*	–	2.7 ± 0.1 × 10^9^	5.7 ± 0.2 × 10^9^	2.3 ± 0.3 × 10^9^	1.2 ± 0.1 × 10^9^
Wild-type	+	5.8 ± 0.3 × 10^6^	3.2 ± 0.1 × 10^6^	1.6 ± 0.1 × 10^6^	3.1 ± 0.1 × 10^5^
Δ*gsp*	+	7.9 ± 0.9 × 10^6^	4.1 ± 0.2 × 10^6^	2.2 ± 0.2 × 10^6^	5.7 ± 0.3 × 10^5^
Δ*sslE*	+	6.3 ± 0.3 × 10^6^	4.1 ± 0.3 × 10^6^	2.1 ± 0.4 × 10^6^	5.0 ± 0.6 × 10^5^

^**a**^ –, no urea present; +, 1.15 M urea present.

^**b**^ Colony-forming units per ml of culture at the indicated time after inoculation, shown as means ± SEM for at least three replicate plate counts.

## Discussion and conclusions

Strains within the species *Escherichia coli* encode different combinations of type II secretion systems, each of which secrete different effectors and presumably provide specific advantageous phenotypes to their host organisms. To this point, the only T2SS shown to be functional in non-pathogenic *E. coli* strains is the chitinase-secreting T2SS_α_, which is the sole T2SS encoded by *E. coli* K-12 [13,14] and whose role in natural environments is unknown. We demonstrate here that, surprisingly, the T2SS_β_ that promotes virulence of the enterotoxic strain H10407 and the enteropathogenic strain E2348/69 is conserved, and secretes a virulence factor homolog, in the non-pathogenic *E. coli* W strain. To our knowledge, this is the first time a virulence-associated type II secretion system has been shown to function in non-pathogenic *E. coli*. Deletion of *sslE* could be complemented *in trans*, indicating that an *sslE* disruption does not prevent expression or assembly of T2SS_β_ in *E. coli* W. We observed that *E. coli* W preferentially secretes SslE under nutrient-rich conditions at human body temperature (37°C), which suggests that SslE may be a colonization factor in non-pathogenic strains. The regulation of SslE secretion in other strains is unclear, but expression of genes encoding the LT-secreting T2SS_β_ in *E. coli* H10407 was also shown to be upregulated at host-associated temperatures [11]. We hope that future experiments will elucidate the role of SslE in host colonization by non-pathogenic *E. coli*.

If secretion of SslE indeed aids diverse *E. coli* in gut colonization, it is perhaps surprising that some gut-derived isolates of *E. coli*, such as K-12 and O157:H7, lack the T2SS responsible for SslE secretion. Such strains may compensate for the loss of biofilm-forming propensity using other mechanisms; strains bearing the F plasmid (such as wild-type K-12) may rely on F pilus-mediated aggregation [15], for example. The genes encoding the SslE-secreting T2SS_β_ are present adjacent to the *pheV* tRNA gene, which appears to be a hypervariable locus in *E. coli*[16-18], so they may be randomly lost at a relatively high rate. Indeed, a comparison between phylogeny and T2SS_α_/T2SS_β_ presence suggests independent losses of T2SS_β_ in non-pathogenic strains (Figure 1). Notably, B and W encode the complete T2SS_β_, while Crook’s and K-12 do not, in spite of the fact that Crook’s diverged from K-12 prior to the divergence of B. This indicates that either Crook’s and K-12 lost the T2SS_β_-encoding genes independently, or that an ancestor of Crook’s, B, and K-12 lost the genes, which were subsequently re-acquired by strain B. An examination of the T2SS_β_-encoding loci in Crook’s and K-12 strongly supports the former explanation. In K-12, the T2SS_β_-encoding *gsp* operon clearly experienced an internal deletion that removed the *gspD-K*_*β*_ genes, inactivating the T2SS. In Crook’s, however, the homologous genomic locus appears entirely different: all *gsp* genes are absent, and in their place is the *fec* operon (encoding a ferric citrate transport system) and a variety of putative ORFs. We infer that the most parsimonious explanation of the phylogenetic distribution of T2SS_β_ is that K-12 and Crook’s both lost the T2SS at different points in their evolutionary histories. It remains an open question what pattern of gene gains and losses best explains the distribution of T2SS_β_ across the diversity of *E. coli* strains not considered in our analysis.

It is of interest to note that a non-polar deletion of the *pppA* gene, encoding a prepilin peptidase, prevents secretion of SslE by *E. coli* W. This result agrees with a similar experiment performed by Strozen et al. to assess effects of PppA on LT secretion in H10407 [12]. Both W and H10407 also encode a second prepilin peptidase (GspO) whose homolog is functional in facilitating ChiA secretion via T2SS_α_ in K-12 [19]. Whether the GspO peptidase is not expressed under conditions associated with SslE secretion in both W and H10407, or whether the two peptidases display different substrate specificities, remains to be determined.

Strikingly, in the presence of the otherwise intact *gsp* operon, deletion of *sslE* was effective in promoting modest urea tolerance. When we first observed the urea-tolerant phenotype of the Δ*gsp* strain, we hypothesized that the mutant’s advantage stemmed from lacking the transmembrane components of the T2SS, particularly the secretin pore in the outer membrane, which might be denatured by urea. The urea tolerance of the Δ*sslE* mutant rules out this hypothesis, however, and indicates that secretion of SslE by T2SS_β_ renders cells modestly more sensitive to urea. Relative urea sensitivity is likely due to indirect effects on cell physiology of bearing surface-displayed SslE or of releasing of SslE into the culture medium.

We report here that enzymatic fusions to the C-terminus of SslE interfere with its targeting to the T2SS, as measured by release of fusion proteins and by display of fusion proteins on the outer leaflet of the outer membrane. Previously, Baldi et al. fused a tetracysteine motif to the C-terminus of E2348/69 SslE and saw that the fusion protein was still displayed on the cell surface [9]. We do not think these results contradict ours, due to the significant structural differences between the fusion proteins in question. We propose that the six amino acids appended to the C-terminus of SslE in the study by Baldi et al. did not affect secretion of SslE, but that our fusions of SslE to large tightly-folded proteins (plant cell wall degrading enzymes from *Cellvibrio japonicus*) occluded important targeting motifs recognized by the T2SS. The uncharacterized nature of T2SS recognition of substrates [20] unfortunately limits our ability to speculate further as to what these motifs might be. Future dissection of the SslE protein with internal deletions and protein fusions may yield new insights into the targeting motif(s) of SslE, and determine whether SslE fusions can be used in the surface display of other proteins.

## Methods

### Growth media, strains and plasmids

*E. coli* strains and plasmids used in this study are summarized in Table 3, and sequences of the plasmids are provided in Additional file 3. The rich (LB) and minimal (Neidhardt MOPS minimal with 0.2% glycerol) media [21,22] contained supplements at the following concentrations: 25 μg/ml kanamycin, 100 μg/ml ampicillin, and 30 μg/ml chloramphenicol. Mutant strains were constructed by replacing various loci with a FRT-*kan*-FRT cassette via the λ Red method, and *kan* cassettes were then removed by FLP excision as described [23,24]. The FRT-*kan*-FRT cassette used for gene disruptions of *gspC-M*, *pppA*, and *sslE* was amplified from Keio mutant genomic DNA [24] using the primer pairs noted in Table 4. To ensure our phenotypes did not result from second-site mutations, we generated all mutant strains twice in parallel and performed assays with two independent isolates, which behaved similarly in all cases.

**Table 3:** Strains and plasmids used in this study

***E. coli *strain or plasmid**	**Description**^**a**^	**Reference or source**^**b**^
Strains		
W	Wild-type *E. coli* W	ATCC 9637
W Δ*gsp*::Kan	W Δ*gspC-M*::FRT-*kan*-FRT	This work
W Δ*gsp*::FRT	W Δ*gspC-M*::FRT, derived by FLP recombination from W Δ*gsp*::Kan	This work
W Δ*pppA*::Kan	W Δ*pppA*::FRT-*kan*-FRT	This work
W Δ*pppA*::FRT	W Δ*pppA*::FRT, derived by FLP recombination from W Δ*pppA*::Kan	This work
W Δ*sslE*::Kan	W Δ*sslE*::FRT-*kan*-FRT	This work
W Δ*sslE*::FRT	W Δ*sslE*::FRT, derived by FLP recombination from W Δ*sslE*::Kan	This work
Plasmids		This work
pRH21	pACYC184-derived; *trc* promoter; *lacI*^q^	This work
pRH31	pTrc99A-derived; *trc* promoter; *lacI*^q^	This work
pMSD6	pRH21 with *sslE* cloned into the MCS	This work
pMSD7	pRH21 with *sslE* lacking the signal peptide-encoding sequence cloned into the MCS	This work
pMSD8	pRH21 with *pppA* cloned into the MCS	This work
pRH153	pRH31 with an *sslE-cel45A* fusion cloned into the MCS	This work
pRH154	pRH31 with an *sslE-pel10A* fusion cloned into the MCS	This work

^**a**^ MCS, multiple cloning site.

^**b**^ ATCC, American Type Culture Collection.

**Table 4:** Primers used in this study

**Name**	**Description**	**Application**
gspKO-up	GACAATCTTTTAATACAGACAAAGAGCATCTGCGAAAAATTGTACGCGGGATTCCGGGGATCCGTCGACC	*gspC-M* deletion
gspKO-dn	CGCCACGTTAACGAGAGTAATTTTATTGATACTAATCTCCTGATACTTTATGTAGGCTGGAGCTGCTTCG	*gspC-M* deletion
pppAKO-dn	ATACACTTGCAGGCCCGCATCCGGCAAGTTACAACAAACAACCTTTAACCATTCCGGGGATCCGTCGACC	*pppA* deletion
pppAKO-dn	TTATTAATAAGAGTTAAAATGTCACTTTGATAATGACGTTGTTATCATTATGTAGGCTGGAGCTGCTTCG	*pppA* deletion
sslEKO-up	TTTCTCCCAGTTACGAATTTTTTAACATTGTTTTGTCACTTGCGTTATTAATTCCGGGGATCCGTCGACC	*sslE* deletion
sslEKO-dn	TTATTTCATGCCGGATGCGGCGTGAACGCCTTATCCGGCATACAGGATTATGTAGGCTGGAGCTGCTTCG	*sslE* deletion
pppA-up	TTATTAGGTACCATGCTTTTTGATGTTTTTCAGC	*pppA* cloning
pppA-dn	ATATTAGGATCCTTAAAACAATGCCTGTAGATAAATTG	*pppA* cloning
sslE-up	TTATTAGGTACCATGAATAAGAAATTTAAATATAAGAAATCG	*sslE* cloning
sslE-noSP-up	TTATTAGGTACCATGTCTTCCTCCGATACG	*sslE-SP* cloning
sslE-dn	TTATTAGGATCCTTACTCGACAGACATCTTATG	*sslE* cloning
sslE-dn-nostop	TTATTAGGATCCGCTCTCGACAGACATCTTATG	*sslE* cloning for fusions
cel45A-noSP-up	TTATTAGGATCCGCAGTTTGTGAATATCGTGTTACC	*sslE-cel45A* fusion
cel45A-dn	TTATTAAAGCTTTTACGGGCAGGTATTACGAATATC	*sslE-cel45A* fusion
pel10A-noSP-up	TTATTAGGATCCGCCTGCAGTTACAAGGTCAC	*sslE-pel10A* fusion
pel10A-dn	TTATTAAAGCTTTTACAGGTAACCCACTTTCTGG	*sslE-pel10A* fusion

All synthetic DNA for plasmid constructions described below was provided by Geneart (Regensburg, Germany). Plasmid pRH21 was constructed from pEG100 [25] by replacing the multiple cloning site (MCS) with a synthetic variant including a tandem His-FLAG tag and by adding the *rrnB*-derived terminators from pTrc99A downstream of the MCS. pRH31 was constructed from pTrc99A [26] by replacing the MCS with the same synthetic variant as in pRH21. pMSD6 was constructed using *sslE* amplified from *E. coli* W genomic DNA with the sslE-up and sslE-dn primers (Table 4). pMSD7 was constructed using *sslE* similarly amplified with the sslE-noSP-up and sslE-dn primers. pMSD8 was constructed using *pppA* similarly amplified with the pppA-up and pppA-dn primers. For construction of pMSD6, pMSD7, and pMSD8, the PCR products were digested with *Acc*65I and *Bam*HI and ligated into the large *Acc*65I/*Bam*HI fragment of pRH21.

For construction of pRH153, *sslE* was amplified from *E. coli* W genomic DNA using primers sslE-up and sslE-dn-nostop, and the PCR product was digested with *Acc*65I and *Bam*HI. A gene encoding the mature form of Cel45A from *Cellvibrio japonicus* Ueda107 was synthesized and codon optimized for *E. coli* expression, then amplified using the cel45A-noSP-up and cel45A-dn primers, and the PCR product was digested with *Bam*HI and *Hind*III. The two digested PCR products (*sslE* and *cel45A*) were ligated into the large *Acc*65I/*Hind*III fragment of pRH31. pRH154 was constructed as pRH153, with a synthetic gene encoding mature Pel10A from *C. japonicus* Ueda107 (with altered codon usage for expression in *E. coli*) being amplified using the pel10A-noSP-up and pel10A-dn primers prior to digestion and ligation.

### Protein expression and detection

For assessing secretion of wild-type SslE, cultures of indicated strains (mutants were all *kan*-marked, except Δ*pppA* mutants, which were unmarked) were grown in liquid media (LB at 37°C unless otherwise noted) with aeration for 16–20 hours. For complementation of the Δ*sslE* mutation, gene expression from plasmids was induced with 1 μM isopropyl-β-d-galactopyranoside (IPTG). Cells were harvested by centrifugation and resuspended in SDS sample buffer (SSB) [21] according to the following formula: resuspension volume (in μl) = 100 × A_600_ × vol harvested (in ml). These concentrated cell lysates were diluted 1:100 in SSB for SDS-PAGE. Cell-free supernatants were concentrated ~10-fold by filtration using Centricon spin columns (Millipore, Billerica, MA, USA), and added to concentrated SSB for SDS-PAGE. Samples were separated on 4-12% SDS-polyacrylamide gels and stained with silver to visualize protein bands [21]. SslE secretion experiments were repeated 2–4 times, and single representative gels are shown.

To produce the images in Figure 2, the stained gels were digitally photographed and gel images were enhanced using Adobe Photoshop software. Linear transformations (contrast and brightness adjustments) were applied to the images for clarity; such transformations were applied uniformly across any given gel image.

### Fusion protein localization by enzyme activity

To measure secretion and surface display of SslE-enzyme fusions, cultures of WT and Δ*pppA*::FRT strains bearing the indicated plasmids were grown in LB at 37°C with aeration for 16–20 hours. Cells were harvested by centrifugation, and cell-free supernatants were removed; an aliquot of collected cells was removed and lysed using the PopCulture reagent from Novagen (Madison, WI, USA). Enzymatic activities associated with intact cells, lysed cells, and cell-free supernatants were then immediately measured. SslE-Cel45A activity was measured using the CRACC assay [27], and SslE-Pel10A activity was measured using the pectate lyase assay described by Collmer [28].

### Growth comparisons

Phenotypic microarray experiments were performed using an OmniLog reader (Biolog, Hayward, CA, USA) as per the manufacturer’s instructions using plate types PM-9 and PM-10. Cultures were grown at 37°C for 48 hours, and respiration data were analyzed using the PM software provided with the OmniLog reader. Strains used were wild-type W and Δ*gsp*::FRT (unmarked deletion of *gspC-M*).

To compare urea tolerances in 96-well plates, wild-type, Δ*gsp*::FRT, and Δ*pppA*::FRT strains were cultured in 200 μl aliquots of LB containing 0, 0.9 M, or 1.15 M urea in 96-well plates (inoculated as 1:100 dilutions from LB overnight cultures). Plates were grown with shaking at 37°C in a Tecan M1000 plate reader (Durham, NC, USA). Growth and survival were followed by regular measurement of A_595_ for each culture.

To compare urea tolerances in glass culture tubes, wild-type, Δ*gsp*::FRT, and Δ*sslE*::FRT strains were cultured in 8 ml volumes of LB containing no urea or 1.15 M urea on a rolling wheel at 37°C. Biological duplicate cultures of each strain were inoculated with 1:1000 dilutions from LB overnight cultures after verification that all overnight cultures grew to equivalent A_600_ turbidity readings. Turbidity in growing cultures was measured by reading A_600_ using a Spectronic 20D digital spectrophotometer; for cultures with high densities (A_600_ > 1.5), aliquots of the culture were diluted 1:10 or 1:20 prior to measurement of A_600_. Viable cells were enumerated by 10-fold serial dilution of cultures into sterile 0.9% NaCl followed by plating of dilutions on non-selective media and colony counting.

### Availability of supporting data

Biolog cultivation data are included as Additional file 1. Data from microtiter plate growth experiments of cells under urea stress are included as in Additional file 2: Figure S1. The sequences of all plasmids described in this study are included as Additional file 3.

## Abbreviations

T2SS: Type II secretion systems; LT: Heat-labile enterotoxin; Gsp: General secretory pathway; WT: Wild-type; MCS: Multiple cloning site.

## Competing interests

The authors declare no competing interests.

## Authors’ contributions

MD, RL, and RH designed experiments and contributed to writing the manuscript. MD and RH performed experiments and analyzed data. All authors read and approved the final manuscript.

## Supplementary Material

Additional file 1 Dye reduction traces for Biolog experiments. Click here for file

Additional file 2: Figure S1 Growth of wild-type and mutant strains with and without urea in 96-well plate experiments. Click here for file

Additional file 3 Sequences of plasmids used in this study. Click here for file

## References

[B1] Korotkov KV Sandkvist M Hol WG The type II secretion system: biogenesis, molecular architecture and mechanism Nat Rev Microbiol 2012 10 336 351 22466878 10.1038/nrmicro2762 PMC3705712

[B2] McLaughlin LS Haft RJF Forest KT Structural insights into the type II secretion nanomachine Curr Opin Struct Biol 2012 22 208 216 10.1016/j.sbi.2012.02.005 22425326 PMC3341957

[B3] Peabody CR Chung YJ Yen MR Vidal-Ingigliardi D Pugsley AP Saier MH Jr Type II protein secretion and its relationship to bacterial type IV pili and archaeal flagella Microbiology 2003 149 3051 3072 10.1099/mic.0.26364-0 14600218

[B4] Hobbs M Mattick JS Common components in the assembly of type 4 fimbriae, DNA transfer systems, filamentous phage and protein-secretion apparatus: a general system for the formation of surface-associated protein complexes Mol Microbiol 1993 10 233 243 10.1111/j.1365-2958.1993.tb01949.x 7934814

[B5] Cianciotto NP Type II secretion: a protein secretion system for all seasons Trends Microbiol 2005 13 581 588 10.1016/j.tim.2005.09.005 16216510

[B6] Sandkvist M Type II secretion and pathogenesis Infect Immun 2001 69 3523 3535 10.1128/IAI.69.6.3523-3535.2001 11349009 PMC98326

[B7] Lathem WW Grys TE Witowski SE Torres AG Kaper JB Tarr PI Welch RA StcE, a metalloprotease secreted by *Escherichia coli* O157:H7, specifically cleaves C1 esterase inhibitor Mol Microbiol 2002 45 277 288 10.1046/j.1365-2958.2002.02997.x 12123444

[B8] Tauschek M Gorrell RJ Strugnell RA Robins-Browne RM Identification of a protein secretory pathway for the secretion of heat-labile enterotoxin by an enterotoxigenic strain of *Escherichia coli* Proc Natl Acad Sci USA 2002 99 7066 7071 10.1073/pnas.092152899 12011463 PMC124529

[B9] Baldi DL Higginson EE Hocking DM Praszkier J Cavaliere R James CE Bennett-Wood V Azzopardi KI Turnbull L Lithgow T The type II secretion system and its ubiquitous lipoprotein substrate, SslE, are required for biofilm formation and virulence of enteropathogenic *Escherichia coli* Infect Immun 2012 80 2042 2052 10.1128/IAI.06160-11 22451516 PMC3370571

[B10] Dunstan RA Heinz E Wijeyewickrema LC Pike RN Purcell AW Evans TJ Praszkier J Robins-Browne RM Strugnell RA Korotkov KV Lithgow T Assembly of the type II secretion system such as found in *Vibrio cholerae* depends on the novel pilotin AspS PLoS Pathog 2013 9 e1003117 10.1371/journal.ppat.1003117 23326233 PMC3542185

[B11] Yang J Baldi DL Tauschek M Strugnell RA Robins-Browne RM Transcriptional regulation of the *yghJ*-*pppA*-*yghG*-*gspCDEFGHIJKLM* cluster, encoding the type II secretion pathway in enterotoxigenic *Escherichia coli* J Bacteriol 2007 189 142 150 10.1128/JB.01115-06 17085567 PMC1797218

[B12] Strozen TG Li G Howard SP YghG (GspS_β_) is a novel pilot protein required for localization of the GspS_β_ type II secretion system secretin of enterotoxigenic *Escherichia coli* Infect Immun 2012 80 2608 2622 10.1128/IAI.06394-11 22585966 PMC3434588

[B13] Archer CT Kim JF Jeong H Park JH Vickers CE Lee SY Nielsen LK The genome sequence of E. coli W (ATCC 9637): comparative genome analysis and an improved genome-scale reconstruction of E. coli BMC Genomics 2011 12 9 10.1186/1471-2164-12-9 21208457 PMC3032704

[B14] Blattner FR Plunkett G Bloch CA Perna NT Burland V Riley M Collado-Vides J Glasner JD Rode CK Mayhew GF The complete genome sequence of *Escherichia coli* K-12 Science 1997 277 1453 1462 10.1126/science.277.5331.1453 9278503

[B15] Lawley TD Wilkins BM Frost L Phillips G, Funnell BE Bacterial conjugation in Gram-negative bacteria Plasmid biology 2004 Washington, D.C: ASM Press 203 226

[B16] Rumer L Jores J Kirsch P Cavignac Y Zehmke K Wieler LH Dissemination of *pheU*- and *pheV*-located genomic islands among enteropathogenic (EPEC) and enterohemorrhagic (EHEC) *E. coli* and their possible role in the horizontal transfer of the locus of enterocyte effacement (LEE) Int J Med Microbiol 2003 292 463 475 10.1078/1438-4221-00229 12635929

[B17] Vimr ER Steenbergen SM Mobile contingency locus controlling *Escherichia coli* K1 polysialic acid capsule acetylation Mol Microbiol 2006 60 828 837 10.1111/j.1365-2958.2006.05158.x 16677296

[B18] Schneider G Dobrindt U Bruggemann H Nagy G Janke B Blum-Oehler G Buchrieser C Gottschalk G Emody L Hacker J The pathogenicity island-associated K15 capsule determinant exhibits a novel genetic structure and correlates with virulence in uropathogenic *Escherichia coli strain* 536 Infect Immun 2004 72 5993 6001 10.1128/IAI.72.10.5993-6001.2004 15385503 PMC517556

[B19] Francetic O Pugsley AP The cryptic general secretory pathway (*gsp*) operon of *Escherichia coli* K-12 encodes functional proteins J Bacteriol 1996 178 3544 3549 8655552 10.1128/jb.178.12.3544-3549.1996 PMC178124

[B20] Filloux A Secretion signal and protein targeting in bacteria: a biological puzzle J Bacteriol 2010 192 3847 3849 10.1128/JB.00565-10 20525826 PMC2916383

[B21] Ausubel FM Brent R Kingston RE Moore DD Seidman JG Smith JA Struhl K Short protocols in molecular biology 2002 5 New York: Wiley

[B22] Neidhardt FC Bloch PL Smith DF Culture medium for enterobacteria J Bacteriol 1974 119 736 747 4604283 10.1128/jb.119.3.736-747.1974 PMC245675

[B23] Thomason L Court DL Bubunenko M Costantino N Wilson H Datta S Oppenheim A Recombineering: genetic engineering in bacteria using homologous recombination Curr Protoc Mol Biol 2007 Chapter 1 Unit 1 1.16.1 1.16.24 10.1002/0471142727.mb0116s78 18265390

[B24] Baba T Ara T Hasegawa M Takai Y Okumura Y Baba M Datsenko KA Tomita M Wanner BL Mori H Construction of *Escherichia coli* K-12 in-frame, single-gene knockout mutants: the Keio collection Mol Syst Biol 2006 2 2006.0008 16738554 10.1038/msb4100050 PMC1681482

[B25] Haft RJ Palacios G Nguyen T Mally M Gachelet EG Zechner EL Traxler B General mutagenesis of F plasmid TraI reveals its role in conjugative regulation J Bacteriol 2006 188 6346 6353 10.1128/JB.00462-06 16923902 PMC1595373

[B26] Amann E Ochs B Abel KJ Tightly regulated *tac* promoter vectors useful for the expression of unfused and fused proteins in *Escherichia coli* Gene 1988 69 301 315 10.1016/0378-1119(88)90440-4 3069586

[B27] Haft RJF Gardner JG Keating DH Quantitative colorimetric measurement of cellulose degradation under microbial culture conditions Appl Microbiol Biotechnol 2012 94 223 229 10.1007/s00253-012-3968-5 22391973

[B28] Collmer A Ried JL Mount MS Assay methods for pectic enzymes Methods Enzymol 1988 161 329 335

