# Supplementary material for: The non-pathogenic Escherichia coli strain W secretes SslE via the virulence-associated type II secretion system beta
Source: BMC Microbiol. 2013 Jun 12;13:130. doi: 10.1186/1471-2180-13-130 (PMC3707838; doi:10.1186/1471-2180-13-130)
Supplement: Additional file 1 — Dye reduction traces for Biolog experiments. [file 1471-2180-13-130-S1.pdf]

Additional file 1: Dye reduction traces for Biolog experiments on plates PM-9 and PM-10.

A document describing the contents of wells in each plate is available from Biolog:

http://www.biolog.com/pdf/pm_lit/PM1-PM10.pdf

Blue = WT, Red = Δ*gsp*::FRT isolate #1, Orange = Δ*gsp*::FRT isolate #2

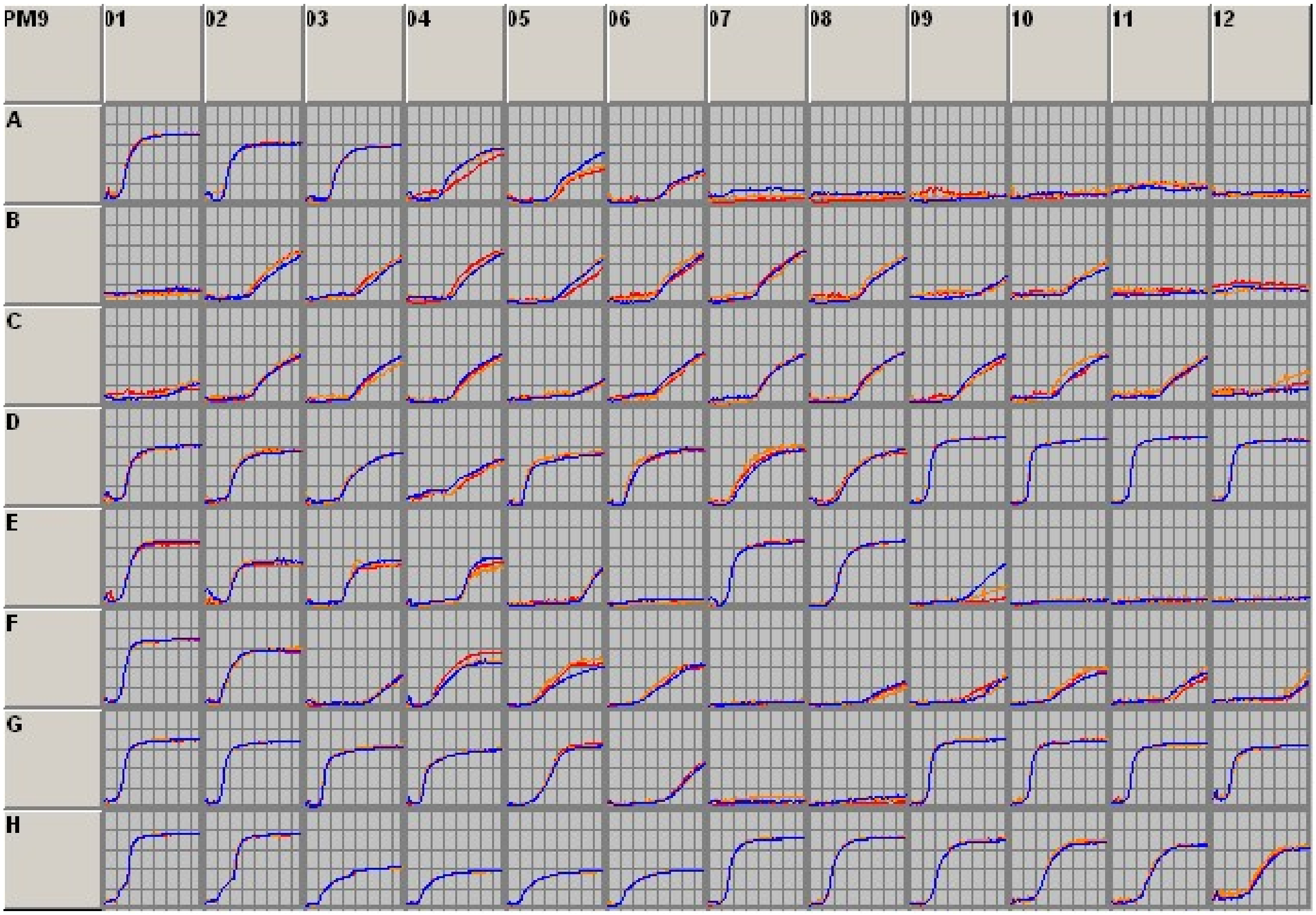

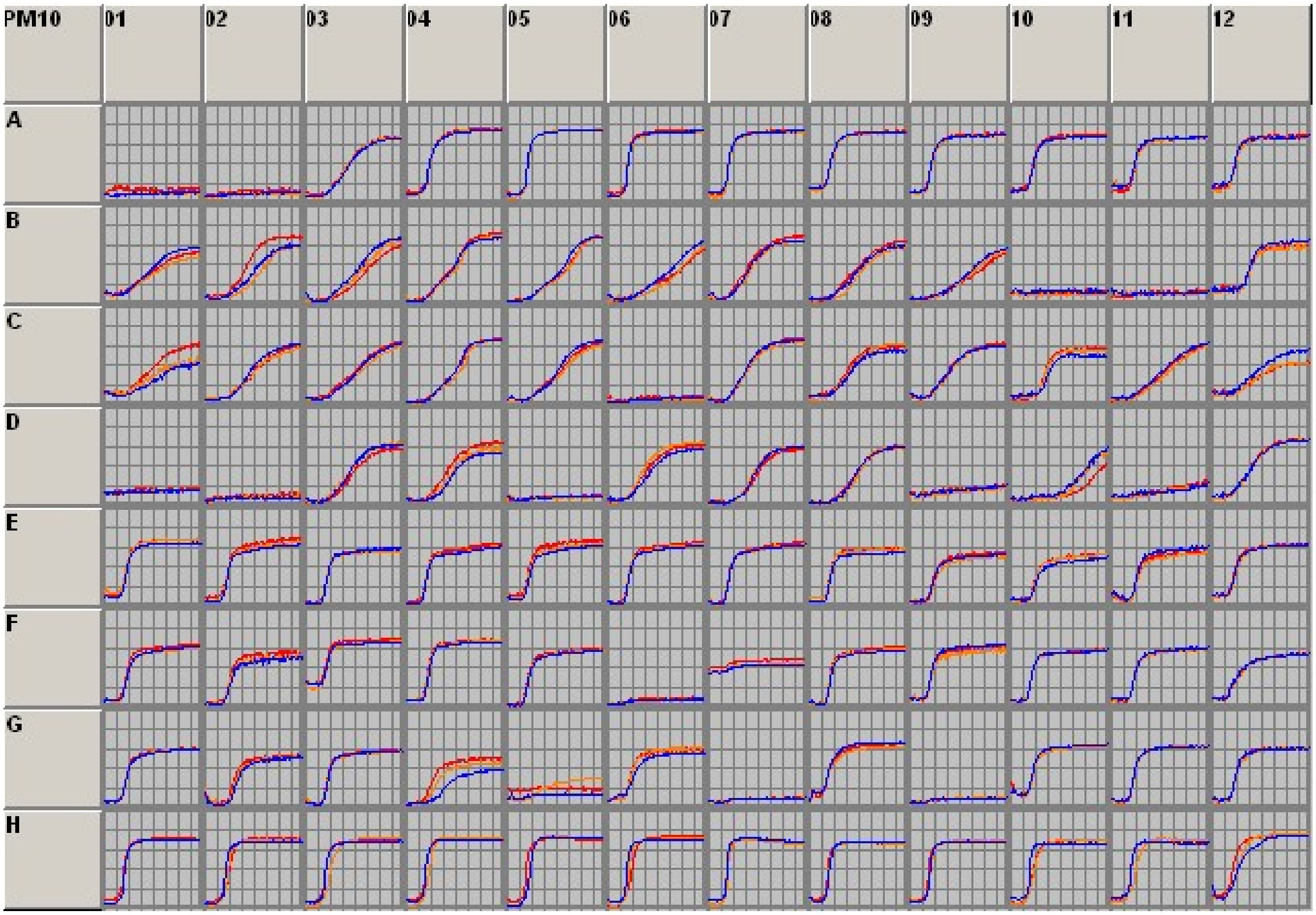
PM10
01
02
03
04
05
06
07
08
09
10
11
12
A
B
C
D
E
F
G
H
