# Supplementary material for: The non-pathogenic Escherichia coli strain W secretes SslE via the virulence-associated type II secretion system beta
Source: BMC Microbiol. 2013 Jun 12;13:130. doi: 10.1186/1471-2180-13-130 (PMC3707838; doi:10.1186/1471-2180-13-130)
Supplement: Additional file 2: Figure S1 — Growth of wild-type and mutant strains with and without urea in 96-well plate experiments. [file 1471-2180-13-130-S2.doc]

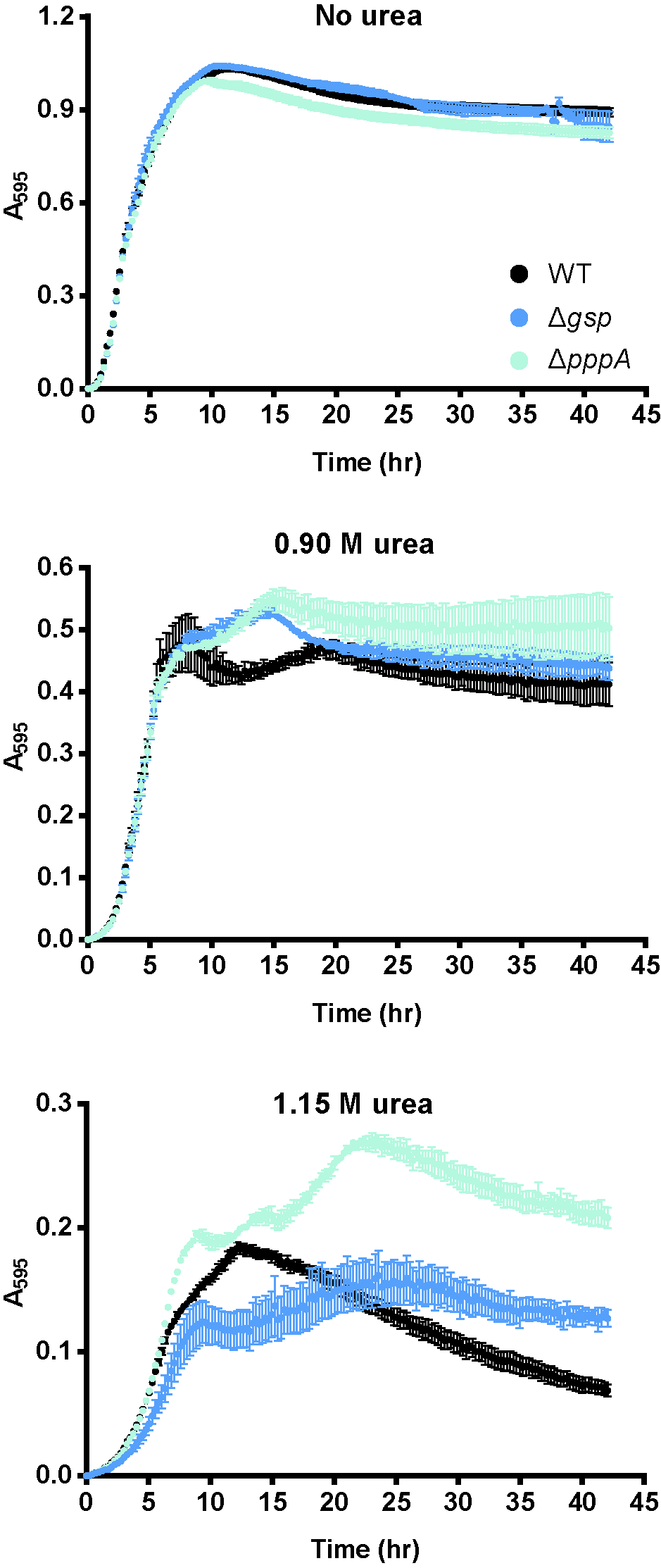


**Figure S1: Mutants lacking *gsp* genes or *pppA* tolerate urea better than their wild-type parent**. Growth curves shown are the mean of 3-4 biological replicates, with ± SEM shown for each data point by vertical bars.
