# Supplementary material for: The non-pathogenic Escherichia coli strain W secretes SslE via the virulence-associated type II secretion system beta
Source: BMC Microbiol. 2013 Jun 12;13:130. doi: 10.1186/1471-2180-13-130 (PMC3707838; doi:10.1186/1471-2180-13-130)
Supplement: Additional file 3 — Sequences of plasmids used in this study. [file 1471-2180-13-130-S3.zip › Plasmid sequence information.pdf]

Additional file 3: sequences of plasmids used in this study

All sequence files are provided in GenBank format. Key genes and features on each plasmid are annotated.

Brief descriptions of each plasmid are given in Table 2 of the text.
